# Mouse Double Minute 2 Homolog-Mediated Ubiquitination Facilitates Forkhead Box P3 Stability and Positively Modulates Human Regulatory T Cell Function

**DOI:** 10.3389/fimmu.2020.01087

**Published:** 2020-06-19

**Authors:** Aiting Wang, Mengdi Yang, Rui Liang, Fangming Zhu, Fuxiang Zhu, Xinnan Liu, Yichao Han, Ruirong Lin, Xiaoxia Wang, Dan Li, Hecheng Li, Xiaojun Yuan, Hui Zhao, Bin Li

**Affiliations:** ^1^Department of Immunology and Microbiology, Shanghai Institute of Immunology, Shanghai Jiao Tong University School of Medicine, Shanghai, China; ^2^Unit of Molecular Immunology, Key Laboratory of Molecular Virology and Immunology, CAS Center for Excellence in Molecular Cell Science, Institut Pasteur of Shanghai, University of Chinese Academy of Sciences, Shanghai, China; ^3^Department of Internal Oncology, Shanghai Jiao Tong University Affiliated Sixth People's Hospital, Shanghai, China; ^4^Shanghai Key Laboratory of Bio-Energy Crops, School of Life Science, Shanghai University, Shanghai, China; ^5^Department of Thoracic Surgery, Ruijin Hospital, Shanghai Jiao Tong University School of Medicine, Shanghai, China; ^6^Department of Biliary-Pancreatic Surgery, Renji Hospital, Shanghai Jiao Tong University School of Medicine, Shanghai, China

**Keywords:** E3 ubiquitin ligase, forkhead box P3 (FOXP3), immunosuppression, mouse double minute 2 homolog (MDM2), regulatory T cell (Treg cell), ubiquitination, monoubiquitination

## Abstract

Regulatory T cells (Treg cells) are essential for maintaining immune tolerance, and the dysfunction of Treg cells may cause autoimmune diseases and tumors. Forkhead box P3 (FOXP3) is the key transcription factor controlling Treg cell development and suppressive function. Mouse double minute 2 homolog (MDM2), an E3 ubiquitin ligase, has been identified as an oncoprotein that mediates the ubiquitination and degradation of tumor suppressor p53; however, whether it has functions in Treg cells remains unknown. Here, we demonstrate that MDM2 positively regulates human Treg cell suppressive function via its mediated ubiquitination and stabilization of FOXP3. Knockdown of MDM2 with shRNA in human primary Treg cells leads to the impaired ability of FOXP3 to regulate the expression levels of downstream genes and the attenuated suppressive capacity of Treg cells, due to FOXP3 instability. Consistently, MDM2 overexpression in human Treg cells enhances FOXP3 stability and Treg cell suppressive capacity. Mechanistically, MDM2 interacts with FOXP3, and mainly mediates monoubiquitination and polyubiquitination of FOXP3, thus stabilizing the protein level of FOXP3. We have also found lysine residues in FOXP3 required for MDM2-mediated ubiquitination. In addition, TCR/CD28 signaling upregulates the expression level of MDM2 and its mediated FOXP3 ubiquitination in human Treg cells. Therefore, our findings reveal that MDM2 in Treg cells could be a potential therapeutic target for treating autoimmune diseases and tumors.

## Introduction

CD4^+^CD25^+^FOXP3^+^ regulatory T cells (Treg cells) are crucial for maintaining immune tolerance and immune homeostasis (1). Impairment or deficiency of immunosuppressive function mediated by Treg cells leads to the occurrence of autoimmune diseases, while excessive immunosuppression promotes the development of tumor (1–4). Forkhead box P3 (FOXP3) is the key transcription factor of Treg cells and plays important roles in Treg cell suppressive function (5–7). CD4^+^CD25^−^ T cells that ectopically expressed FOXP3 acquired Treg cell phenotypes, because they exhibited suppressive activity, and could inhibit interleukin-2 (IL-2) expression while facilitating the expression of CD25 and cytotoxic T-lymphocyte antigen 4 (CTLA-4) (5, 6). Studies have established that FOXP3 could be regulated at the level of post-translational modifications, which include acetylation, phosphorylation, poly(ADP-ribosyl)ation, arginine methylation, and ubiquitination, affecting the stability and function of FOXP3 and Treg cells (8–20). In recent years, it has been reported that several E3 ubiquitin ligases or deubiquitinases (DUBs) modulate the K48-linked polyubiquitination of FOXP3, thus impacting FOXP3 stability and Treg cell function (17–19). However, other types of ubiquitination of FOXP3 protein are largely unclear. Due to the distinct sites and types, ubiquitination not only causes protein degradation, but also stabilizes the function of substrates (21, 22); therefore, other enzymes that regulate different types of FOXP3 ubiquitination remain to be identified.

Mouse double minute 2 homolog (MDM2) is an E3 ubiquitin ligase containing RING finger domain (23). It has been identified as an oncoprotein that mediates the ubiquitination and degradation of tumor suppressor p53 (24). MDM2 controls the expression level of p53 through two mechanisms: firstly, p53 degradation is promoted by MDM2 ubiquitin ligase activity. Secondly, the transcription activation of p53 is inhibited by MDM2 (25–27). Although the most widely studied function of MDM2 is to inhibit p53 activity, studies have revealed that there are other substrates of MDM2 in cells that may contribute to MDM2-mediated regulation of cell fate and function in cancer (24). For example, MDM2 inhibits the cell cycle stagnation mediated by Retinoblastoma (Rb) protein through the degradation of Rb protein (28). MDM2 can also promote cell proliferation via mediating ubiquitination and degradation of p21 and hnRNPK proteins (28). In addition, MDM2-mediated FOXO3a degradation can inhibit FOXO3a-mediated apoptosis during tumor development (29). All the above proteins are regulated by MDM2-mediated K48-linked polyubiquitination and degradation; however, MDM2-mediated non-degradative ubiquitination remains unclear. Despite the fact that MDM2 has been widely studied in tumors, little is known about whether MDM2 can regulate the function of the immune system. Recently, it has been demonstrated that MDM2 negatively regulates T cell activation, which reveals the critical role of MDM2 in effector T cells (Teff cells) (30). However, it remains unknown whether MDM2 may modulate the function of Treg cells.

Here, we made efforts to investigate whether MDM2 was indispensable for human Treg cell function and the underlying mechanisms. We found that MDM2 enhanced the stability of FOXP3 protein through mediating various types of FOXP3 ubiquitination, especially monoubiquitination, therefore positively regulating human Treg cell function. We also identified the sites on FOXP3 protein responsible for MDM2-mediated ubiquitination of FOXP3. Furthermore, TCR/CD28 signaling could upregulate the expression level of MDM2 and its mediated FOXP3 ubiquitination in human Treg cells. Our findings that MDM2 positively regulates the immunosuppressive activity of Treg cells provide new insights into antitumor therapy via inhibiting MDM2 in Treg cells.

## Results

### MDM2 Is Crucial for Human Treg Cell Function

**Figure 1:**
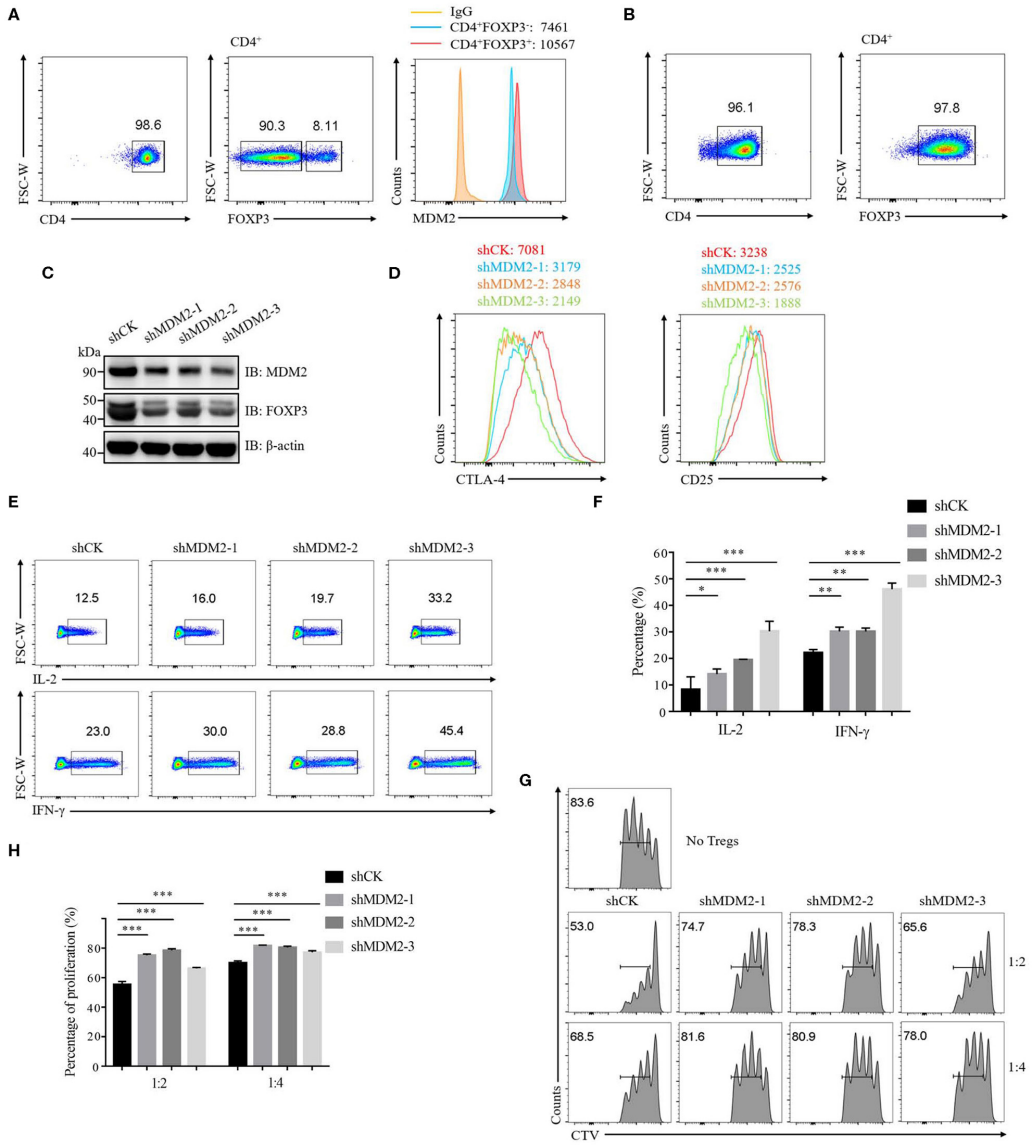
MDM2 is crucial for human Treg cell function. **(A)** The expression level of MDM2 was detected in Treg cells (CD4^+^FOXP3^+^) and Tconv cells (CD4^+^FOXP3^−^) of CD4^+^ T cells isolated from healthy donor PBMC, as indicated by MFI. **(B)** Naïve CD4^+^ T cells (CD4^+^CD25^low^CD127^high^CD45RA^+^) from healthy donors were differentiated into iTreg cells *in vitro* in the presence of anti-CD3/CD28 beads, IL-2 (100 U/ml), and TGF-β (5 ng/ml), and the differentiation efficiency was measured after 7 days, as indicated by the percentage of FOXP3^+^. Human iTreg cells were used for a series of experiments 7 days post-differentiation. **(C)** MDM2 knockdown assay was performed in human iTreg cells using MDM2 shRNA-bearing lentiviruses, and the expression levels of MDM2 and FOXP3 were examined by Western blot. **(D)** MDM2 knockdown assay was performed in human iTreg cells, followed by the analysis of CTLA-4 and CD25 expression. **(E)** IL-2 and IFN-γ production from human iTreg cells were assessed after knockdown of MDM2 by lentiviruses carrying MDM2 shRNA. **(F)** The percentages of IL-2 and IFN-γ production from human iTreg cells after MDM2 knockdown were analyzed as demonstrated in **(E)**. **(G)** *In vitro* suppression assay was performed in human iTreg cells infected by shCK or MDM2 shRNA-bearing lentiviruses. **(H)** The proportions of Teff cell (CD4^+^CD127^high^CD25^low^) proliferation were analyzed as demonstrated in **(G)** (*n* = 3). Error bars represent mean ± S.D. *p < 0.05, **p < 0.01, ***p < 0.001.

To investigate whether MDM2 plays an essential role in human primary Treg cells, we first detected the expression of MDM2 in Treg cells and conventional T cells (Tconv cells). Compared with Tconv cells, the expression level of MDM2 was higher in Treg cells, as indicated by mean fluorescence intensity (MFI) (Figure 1A), suggesting that MDM2 may be required for Treg cells. Then, human-induced Treg cells (iTreg cells) were differentiated from naïve CD4^+^ T cells *in vitro* (Figure 1B) and were used to explore the effects of MDM2 on human Treg cell function by MDM2 knockdown assay. A significantly decreased level of MDM2 expression was observed in Treg cells after MDM2 knockdown, accompanied by the attenuated expression of FOXP3 (Figure 1C), implying a correlation between MDM2 and FOXP3. MFI of CTLA-4 and CD25, which are Treg cell signature molecules directly regulated by FOXP3 (31, 32), was reduced in Treg cells after knockdown of MDM2 (Figure 1D). Furthermore, Treg cells with MDM2 knockdown produced more IL-2 and interferon-γ (IFN-γ) (Figures 1E,F), indicating the transition from Treg cells to Teff cells, especially type 1 helper T cells (Th1 cells). However, there was no obviously altered cytokine production from Teff cells with MDM2 knockdown, compared to those intact Teff cells (Supplementary Figure 1), suggesting that IL-2 and IFN-γ regulation by MDM2 depends on FOXP3 due to the lack of FOXP3 expression in Teff cells. Meanwhile, the impact of MDM2 knockdown on the function of human Treg cells was examined by *in vitro* suppression assay. Treg cells with MDM2 knockdown displayed markedly impaired capacity of suppressing Teff cell proliferation (Figures 1G,H). These findings implicate that MDM2 knockdown in human Treg cells leads to impaired Treg cell function and acquisition of Teff cell phenotypes; therefore, MDM2 is crucial for human Treg cell suppressive function.

### MDM2 Positively Modulates Human Treg Cell Function

**Figure 2:**
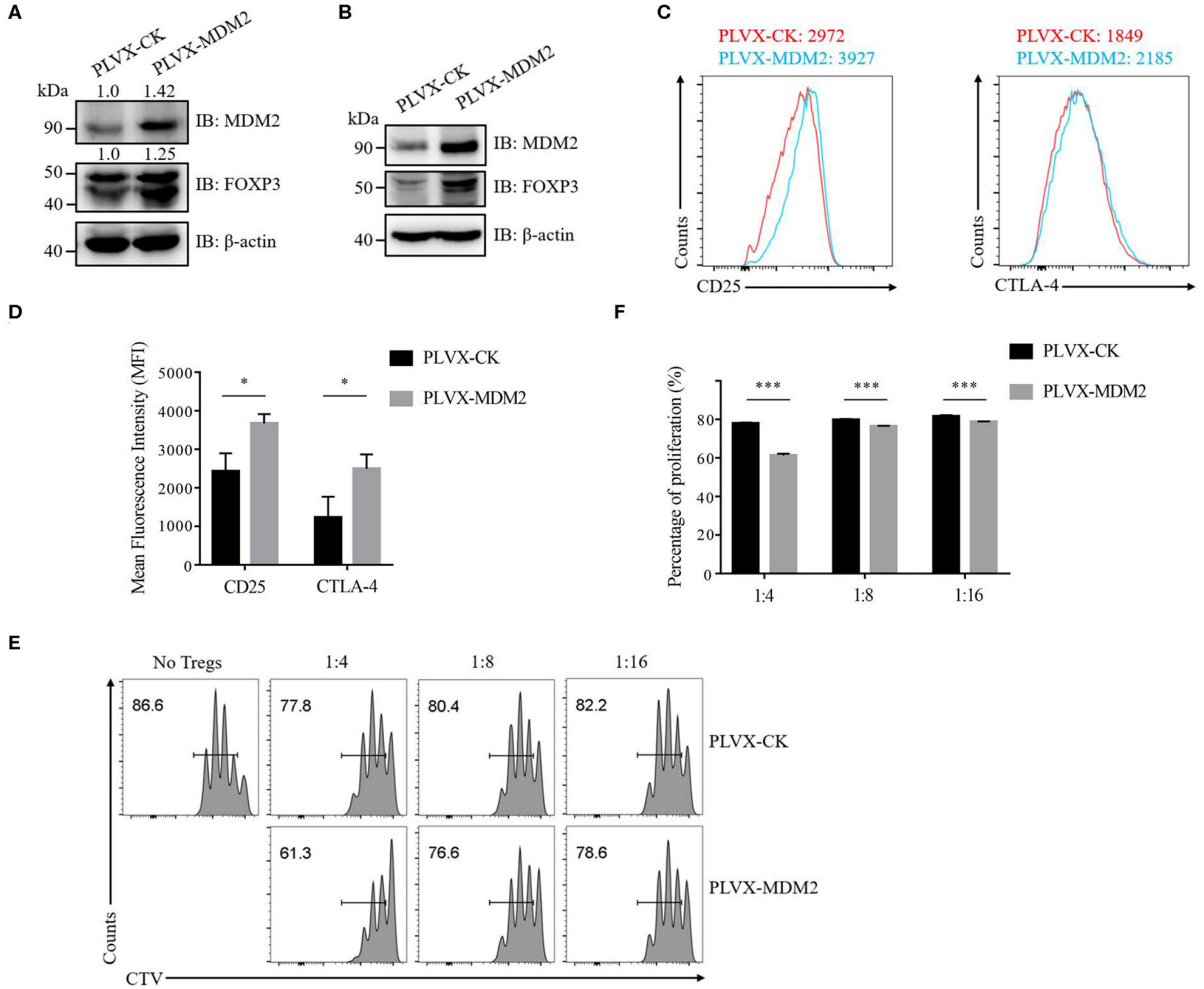
MDM2 positively modulates human Treg cell function. **(A)** The expression levels of MDM2 and FOXP3 were examined in human iTreg cells infected by lentiviruses carrying PLVX-CK-GFP or PLVX-MDM2-GFP constructs. **(B)** MDM2 overexpression assay was performed in Jurkat T cells with stable expression of Flag-FOXP3 using lentiviruses, followed by the detection of MDM2 and FOXP3 expression by Western blot. **(C)** CD25 and CTLA-4 expression was assessed in human iTreg cells after MDM2 overexpression. **(D)** The expression levels of CD25 and CTLA-4 in human iTreg cells after MDM2 overexpression were analyzed as demonstrated in **(C)**. **(E)** *In vitro* suppression assay was performed in human iTreg cells with or without MDM2 overexpression. **(F)** The proportions of Teff cell proliferation were analyzed as demonstrated in **(E)** (*n* = 3). Error bars represent mean ± S.D. *p < 0.05, ***p < 0.001.

We then further examined the importance of MDM2 in human Treg cells by performing MDM2 overexpression assay. Following MDM2 overexpression, the expression levels of MDM2 and FOXP3 were upregulated in both human Treg cells (Figure 2A) and Jurkat T cells with stable expression of Flag-tagged FOXP3 (Figure 2B), indicating a positive correlation between MDM2 and FOXP3 expression, which is consistent with our MDM2 knockdown data. We also detected the impact of MDM2 overexpression on CD25 and CTLA-4 expression in human Treg cells, and observed that Treg cells overexpressing MDM2 exhibited higher MFI of CD25 and CTLA-4 (Figures 2C,D). What is more, Treg cells with MDM2 overexpression were significantly more capable of inhibiting Teff cell proliferation, examined by *in vitro* suppression assay (Figures 2E,F). These results suggest that MDM2 in human Treg cells positively regulates the expression of Treg cell signature genes and Treg cell function.

### MDM2 Stabilizes the Protein Level of Human FOXP3

**Figure 3:**
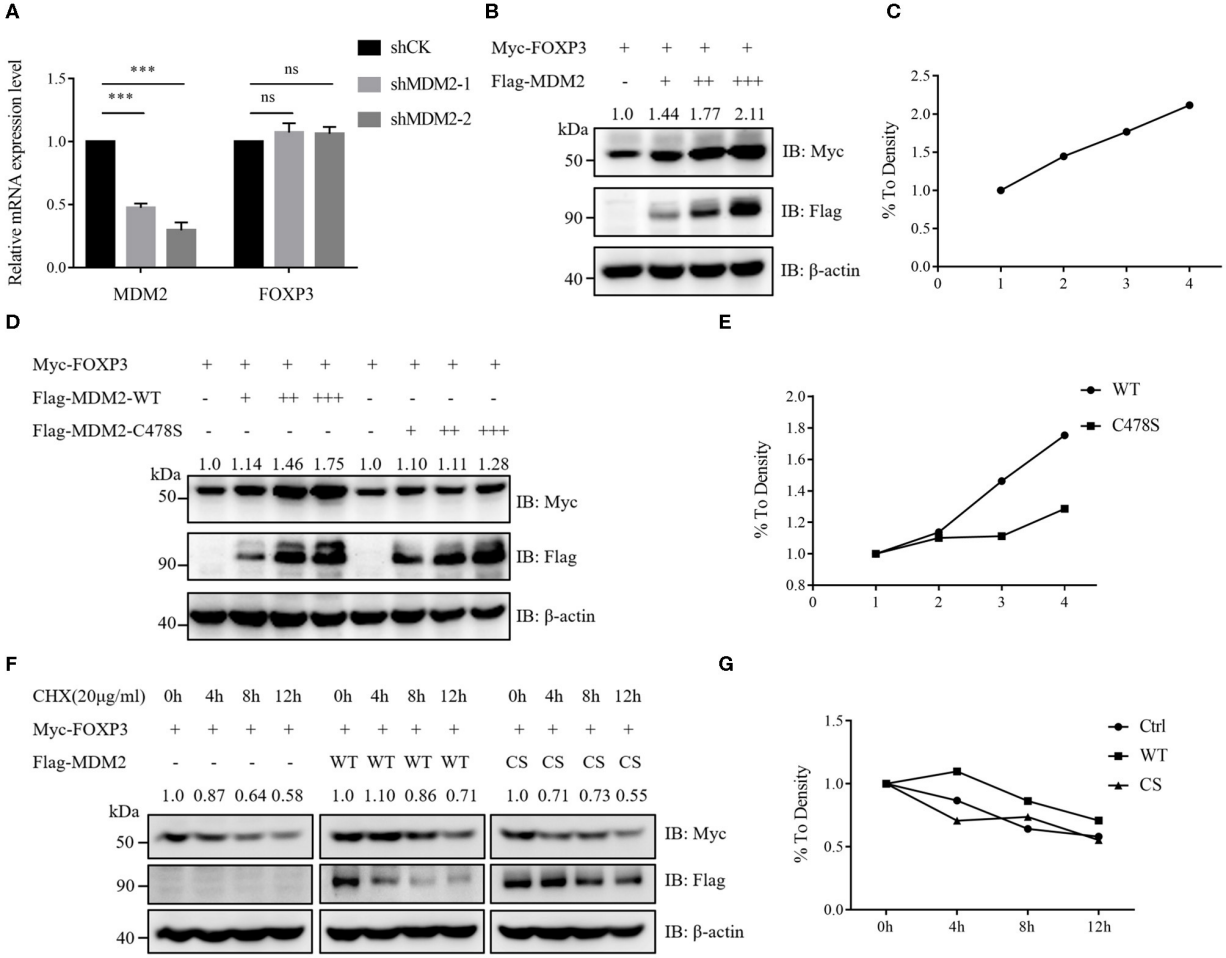
MDM2 stabilizes the protein level of human FOXP3. **(A)** MDM2 knockdown assay was performed in human iTreg cells using MDM2 shRNA-carrying lentiviruses, and the mRNA expression levels of MDM2 and FOXP3 were examined by qRT-PCR (*n* = 3). Error bars represent mean ± S.D. ***p < 0.001; ns, not significant. **(B,C)** Myc-tagged FOXP3 and doses of Flag-tagged MDM2 were co-expressed in HEK293T cells, and Myc-FOXP3 expression was detected 48 h post-transfection by Western blot. **(D,E)** Myc-tagged FOXP3 and doses of Flag-tagged wild-type (WT) MDM2 or C478S MDM2 were co-transfected into HEK293T cells, followed by Western blot analysis for Myc-FOXP3 expression after 48 h. **(F,G)** HEK293T cells were co-transfected with Myc-tagged FOXP3 and Flag vector, Flag-tagged WT MDM2, or C478S MDM2, and were treated with the protein synthesis inhibitor cycloheximide (CHX) for 0, 4, 8, or 12 h before harvesting, followed by the detection of Myc-FOXP3 half-life. The above data are derived from more than three independent experiments.

Our results of MDM2 knockdown and overexpression assays demonstrated that MDM2 could positively modulate Treg cell function and there was a positive correlation between MDM2 and FOXP3, prompting us to hypothesize that MDM2 may regulate the expression level of human FOXP3. Human Treg cells with MDM2 knockdown or overexpression displayed downregulated or upregulated FOXP3 expression, respectively (Figures 1C, 2A). Nevertheless, the transcription level of *FOXP3* gene was comparable in Treg cells with or without MDM2 knockdown (Figure 3A), indicating that MDM2 may regulate FOXP3 expression in human Treg cells through post-translational modification rather than transcriptional regulation.

To further determine how MDM2 modulates the stability of human FOXP3 protein, we examined the effects of MDM2 on FOXP3 stability in HEK293T cell line. Myc-tagged FOXP3 and Flag-tagged MDM2 were co-expressed in HEK293T cells, and it was observed that the protein level of FOXP3 was upregulated along with the increasing dose of MDM2, implying that MDM2 could stabilize the protein level of FOXP3 in a dose-dependent manner (Figures 3B,C). To investigate whether the ability of MDM2 to stabilize FOXP3 protein depends on its E3 ubiquitin ligase activity, we constructed the enzyme-inactive mutant of MDM2 C478S, and co-transfected Myc-tagged FOXP3 and Flag-tagged wild-type (WT) MDM2 or C478S MDM2 into HEK293T cells. We found that C478S mutant failed to stabilize the expression level of FOXP3 protein, compared to WT MDM2 (Figures 3D,E). To further verify the above results, HEK293T cells were co-transfected with Myc-tagged FOXP3 and Flag vector, Flag-tagged WT MDM2, or C478S MDM2, and were treated with the protein synthesis inhibitor cycloheximide (CHX). WT MDM2 overexpression prolonged the half-life of FOXP3 protein and inhibited the degradation of FOXP3 protein, whereas MDM2 C478S mutant had no obvious effect (Figures 3F,G). Taken together, these findings suggest that MDM2 promotes the stability of FOXP3 protein and its E3 ubiquitin ligase activity is critical for MDM2-mediated stabilization of FOXP3.

### MDM2 Interacts and Colocalizes With FOXP3

**Figure 4:**
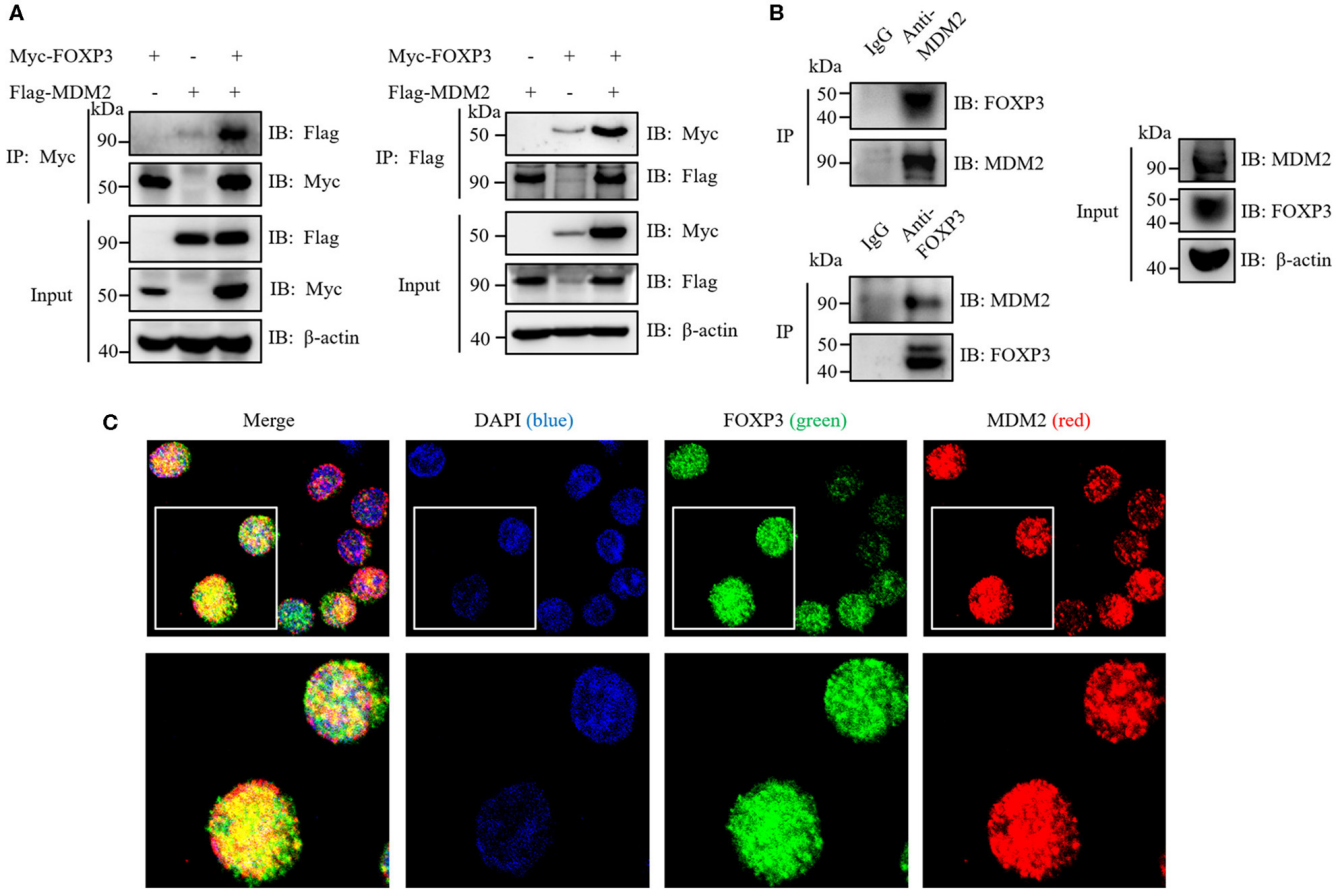
MDM2 interacts and colocalizes with FOXP3. **(A)** HEK293T cells were co-transfected with Myc-tagged FOXP3 and Flag-tagged MDM2, and were harvested after 48 h for co-immunoprecipitation (co-IP) assay with anti-Myc or anti-Flag antibodies. **(B)** Human iTreg cells were harvested for endogenous IP assay with anti-MDM2 or anti-FOXP3 antibodies. **(C)** Confocal assay was performed in human iTreg cells with DAPI (blue), anti-FOXP3 (green), and anti-MDM2 (red) antibodies. The above data are derived from more than three independent experiments.

To investigate whether MDM2 mediates the direct ubiquitination and stabilization of FOXP3, we first detected whether MDM2 could interact with FOXP3. Myc-tagged FOXP3 and Flag-tagged MDM2 were overexpressed in HEK293T cells, followed by reciprocal immunoprecipitation assay with anti-Myc or anti-Flag antibodies. The results demonstrated that MDM2 associated with FOXP3 under ectopic expression conditions in HEK293T cells (Figure 4A). In addition, an interaction between MDM2 and FOXP3 was observed in human Treg cells by endogenous immunoprecipitation assay with anti-MDM2 or anti-FOXP3 antibodies (Figure 4B). Confocal assay demonstrated that in human Treg cells, FOXP3 mainly colocalized with MDM2 in the nucleus (Figure 4C). Thus, MDM2 interacts and colocalizes with FOXP3 in human primary Treg cells.

### MDM2 Mediates Ubiquitination of FOXP3, Which Facilitates FOXP3 Stability

**Figure 5:**
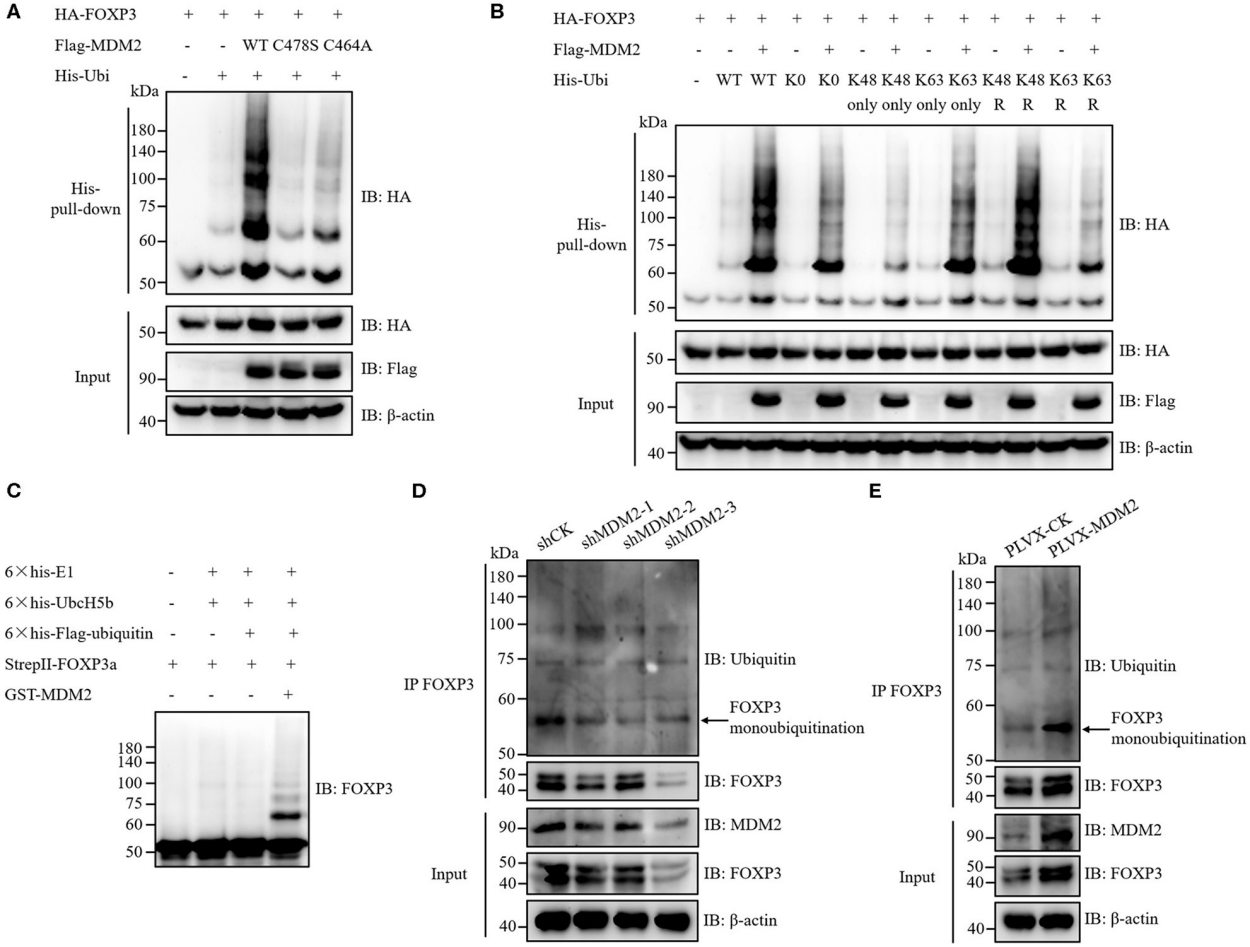
MDM2 mediates ubiquitination of FOXP3, which facilitates FOXP3 stability. **(A)** HA-tagged FOXP3, His-tagged ubiquitin (Ubi), Flag-tagged WT MDM2, and Flag-tagged MDM2 catalytically inactive mutant C478S or C464A were co-transfected into HEK293T cells. Forty-eight hours post-transfection, His-pull-down assay and Western blot assay were performed to examine the levels of HA-FOXP3 ubiquitination. **(B)** HA-tagged FOXP3, Flag-tagged MDM2, and His-tagged WT ubiquitin, or ubiquitin mutants (K0: all lysine residues were mutated into arginine resides; K48 only or K63 only: only K48 or K63 lysine residue remained; K48R or K63R: K48 or K63 lysine was mutated into arginine) were co-transfected into HEK293T cells. Forty-eight hours post-transfection, the levels of HA-FOXP3 ubiquitination were assessed by His-pull-down assay and Western blot assay. **(C)** 6 × his-E1 (400 ng), 6 × his-UbcH5b (E2) (800 ng), 6 × his-Flag-ubiquitin (12 μg), StrepII-FOXP3a (400 ng), and GST-MDM2 (1.5 μg) were incubated at 37°C in a cell-free system for 2 h, followed by termination with SDS loading buffer. FOXP3 ubiquitination was examined by Western blot assay with anti-FOXP3 antibodies. **(D)** FOXP3 ubiquitination was detected in human Treg cells infected with lentiviruses carrying MDM2 shRNA or shCK, by using immunoprecipitation assay and Western blot assay. The arrow indicates FOXP3 monoubiquitination. **(E)** FOXP3 ubiquitination was checked in human Treg cells with or without MDM2 overexpression. The arrow indicates FOXP3 monoubiquitination. The above data are derived from more than three independent experiments.

Given that MDM2 interacts with FOXP3 and its E3 ubiquitin ligase activity is required for stabilization of FOXP3, we hypothesized that MDM2 mediated the ubiquitination of FOXP3, thus facilitating the stability of FOXP3. To verify this hypothesis, HA-tagged FOXP3, His-tagged ubiquitin (Ubi), and Flag-tagged WT MDM2 or its enzyme-inactive mutant C478S or C464A were co-transfected into HEK293T cells, followed by His-pull-down assay. As expected, WT MDM2, but neither MDM2 C478S nor MDM2 C464A, could mediate the ubiquitination modification and stabilization of FOXP3 protein (Figure 5A).

According to the ubiquitin linkage types on the substrates, several ubiquitination forms have been identified: monoubiquitination; Lys6-, Lys11-, Lys27-, Lys29-, Lys33-, Lys48-, or Lys63-linked polyubiquitination; Met1-linked linear polyubiquitination; and mixed-linkage polyubiquitination (33, 34). Different types of ubiquitin linkage endow ubiquitination with distinct functions. For instance, Lys48-linked polyubiquitination mediates the degradation of target proteins in a proteasome-dependent mechanism, whereas Lys63-linked polyubiquitination and monoubiquitination can stabilize target proteins at post-translational level (20, 34, 35). To determine which types of ubiquitination on FOXP3 are mediated by MDM2, HA-tagged FOXP3 and Flag-tagged MDM2 were co-expressed with several His-tagged ubiquitin mutants in HEK293T cells, followed by His-pull-down assay. Obvious MDM2-mediated ubiquitination of FOXP3 was observed with ubiquitin K0 mutant, in which all of the seven lysine residues in ubiquitin were replaced with arginine residues, suggesting that MDM2 could catalyze FOXP3 monoubiquitination (Figure 5B). More MDM2-mediated ubiquitination of FOXP3 was observed with ubiquitin K63 only mutant (only K63 lysine remained), compared with K48 only mutant. Consistently, less MDM2-mediated ubiquitination of FOXP3 occurred with K63R mutant (only K63 lysine was mutated into arginine), compared to K48R mutant. These data indicate that MDM2 can ubiquitinate FOXP3 through K63 linkage (Figure 5B). Likewise, we found that MDM2 could also catalyze Lys29- and Lys33-linked polyubiquitination of FOXP3 (Supplementary Figures 2A,B). Therefore, MDM2 mainly mediates monoubiquitination of FOXP3, as well as K63-, K29-, and K33-linked polyubiquitination.

To determine whether the ubiquitination modification of FOXP3 is mediated by MDM2 directly, we performed *in vitro* ubiquitination assay by using recombinant GST-MDM2 (purchased from R&D systems), 6 × his-E1, 6 × his-UbcH5b (E2), 6 × his-Flag-ubiquitin, and StrepII-FOXP3a, which were purified previously (17, 20). The result demonstrated that MDM2 could ubiquitinate FOXP3 directly in this cell-free system, especially through monoubiquitination (Figure 5C).

We next investigated the influence of MDM2 on mediating FOXP3 ubiquitination in human Treg cells. After MDM2 knockdown with shRNA-bearing lentiviruses, the level of FOXP3 ubiquitination, especially monoubiquitination, together with FOXP3 expression, was attenuated in both human primary Treg cells and Jurkat T cells with stable expression of Flag-FOXP3 (Figure 5D and Supplementary Figure 2C), indicating that the ubiquitination and stabilization of FOXP3 protein in these cells was facilitated by the endogenous MDM2. Consistently, the level of FOXP3 ubiquitination (especially monoubiquitination) and FOXP3 expression level were elevated in human Treg cells overexpressing MDM2 (Figure 5E). Taken together, MDM2 catalyzes various types of ubiquitination of FOXP3, especially monoubiquitination, thus facilitating the stability of FOXP3.

### Ten Key Lysine Residues in FOXP3 Are Responsible for MDM2-Mediated Ubiquitination

**Figure 6:**
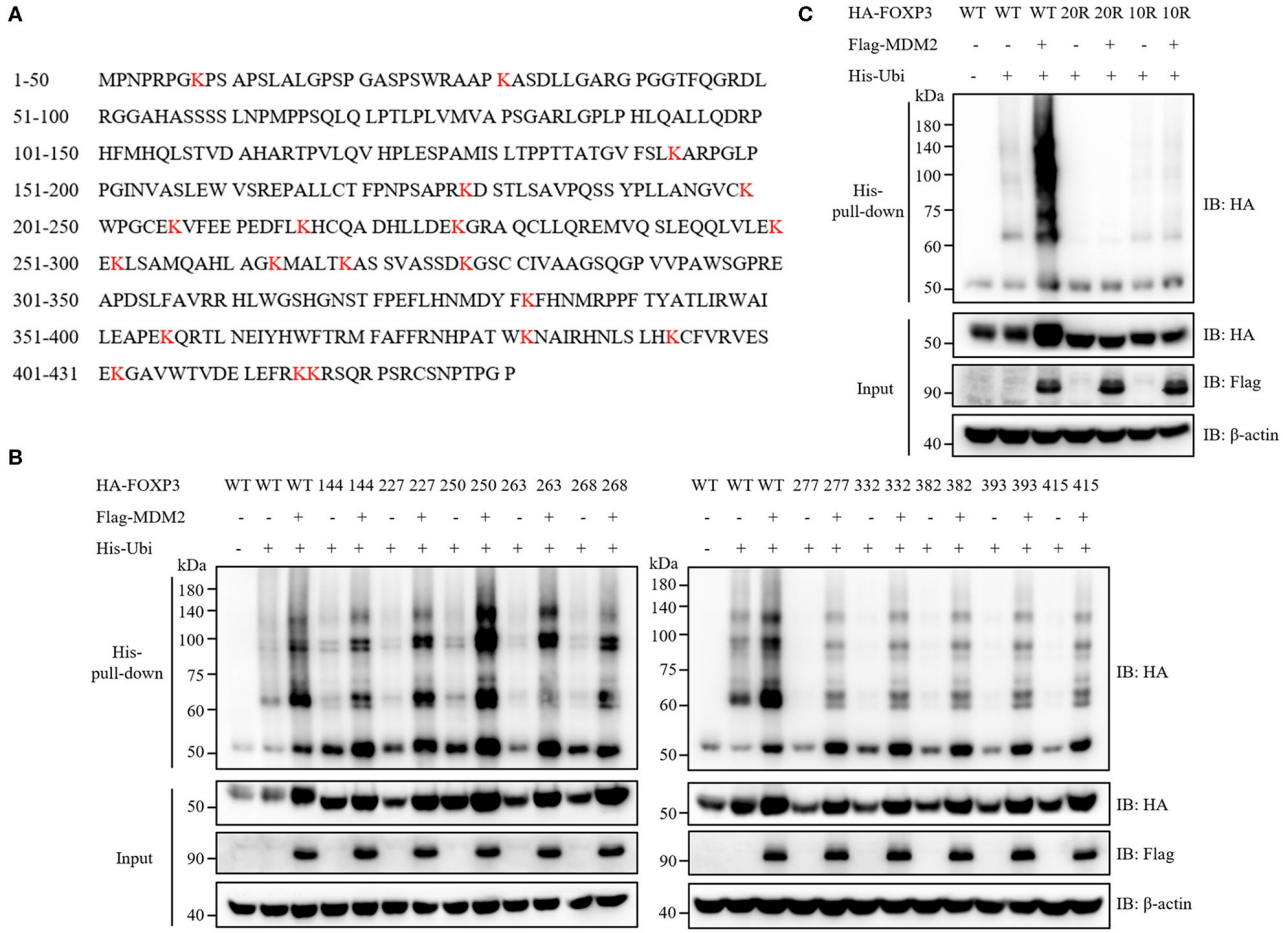
Ten key lysine residues in FOXP3 are responsible for MDM2-mediated ubiquitination. **(A)** The amino acid sequence of human FOXP3 full-length protein was searched from NCBI Protein Database. Human FOXP3 protein contains 20 lysine (K) residues, which are marked in red. **(B)** A series of FOXP3 mutants (in which only one lysine residue remained) were utilized to explore the lysine residues in FOXP3 responsible for MDM2-mediated ubiquitination. HA-tagged WT FOXP3 or FOXP3 mutants, Flag-tagged MDM2, and His-tagged ubiquitin were co-expressed in HEK293T cells, and 48 h after transfection, His-pull-down assay and Western blot assay were performed to assess the ubiquitination levels of HA-FOXP3. Ten FOXP3 mutants (K144 only, K227 only, K250 only, K263 only, K268 only, K277 only, K332 only, K382 only, K393 only, and K415 only) could be ubiquitinated and stabilized by MDM2. **(C)** HA-tagged WT FOXP3, FOXP3 20R mutant (in which all lysine residues were replaced with arginine residues) or FOXP3 10R mutant (in which the above 10 lysine residues were mutated into arginine residues), Flag-tagged MDM2, and His-tagged ubiquitin were co-transfected into HEK293T cells, and 48 h post-transfection, His-pull-down assay and Western blot assay were performed to detect the ubiquitination levels of HA-FOXP3. The above data are derived from more than three independent experiments.

We then examined which sites of FOXP3 could be ubiquitinated by MDM2. Ubiquitination modification usually occurs on the lysine residue of target protein (33), and there are 20 lysine residues in the full-length FOXP3 protein (Figure 6A). We utilized a series of FOXP3 mutants previously constructed (17) to explore the lysine residues in FOXP3 responsible for MDM2-mediated ubiquitination. All lysine residues in WT FOXP3 were replaced with arginine residues to generate FOXP3 20R mutant, and then the reverse mutation with single lysine was performed to produce a single lysine mutation on the basis of FOXP3 20R. For example, FOXP3 K8 only mutant represented that only lysine 8 in FOXP3 remained, and all of other 19 lysine residues became arginine. Co-expression and His-pull-down assay demonstrated that 10 FOXP3 mutants (FOXP3 K144 only, K227 only, K250 only, K263 only, K268 only, K277 only, K332 only, K382 only, K393 only, and K415 only) could be ubiquitinated and stabilized by MDM2 (Figure 6B), but 10 other mutants could not (Supplementary Figure 3). To further determine the requirement of the 10 lysine residues in FOXP3 for MDM2-mediated ubiquitination and stabilization of FOXP3, we constructed FOXP3 10R mutant in which the 10 lysine residues were mutated into arginine residues, and performed co-expression and His-pull-down assay. MDM2-mediated ubiquitination and stabilization was observed with WT FOXP3, but not with FOXP3 10R or FOXP3 20R mutant (Figure 6C). Therefore, these findings reveal that MDM2 mediates the ubiquitination of FOXP3 on lysine K144, K227, K250, K263, K268, K277, K332, K382, K393, and K415.

### TCR/CD28 Signaling Enhances MDM2 Expression and MDM2-Mediated Ubiquitination of FOXP3 in Human Treg Cells

**Figure 7:**
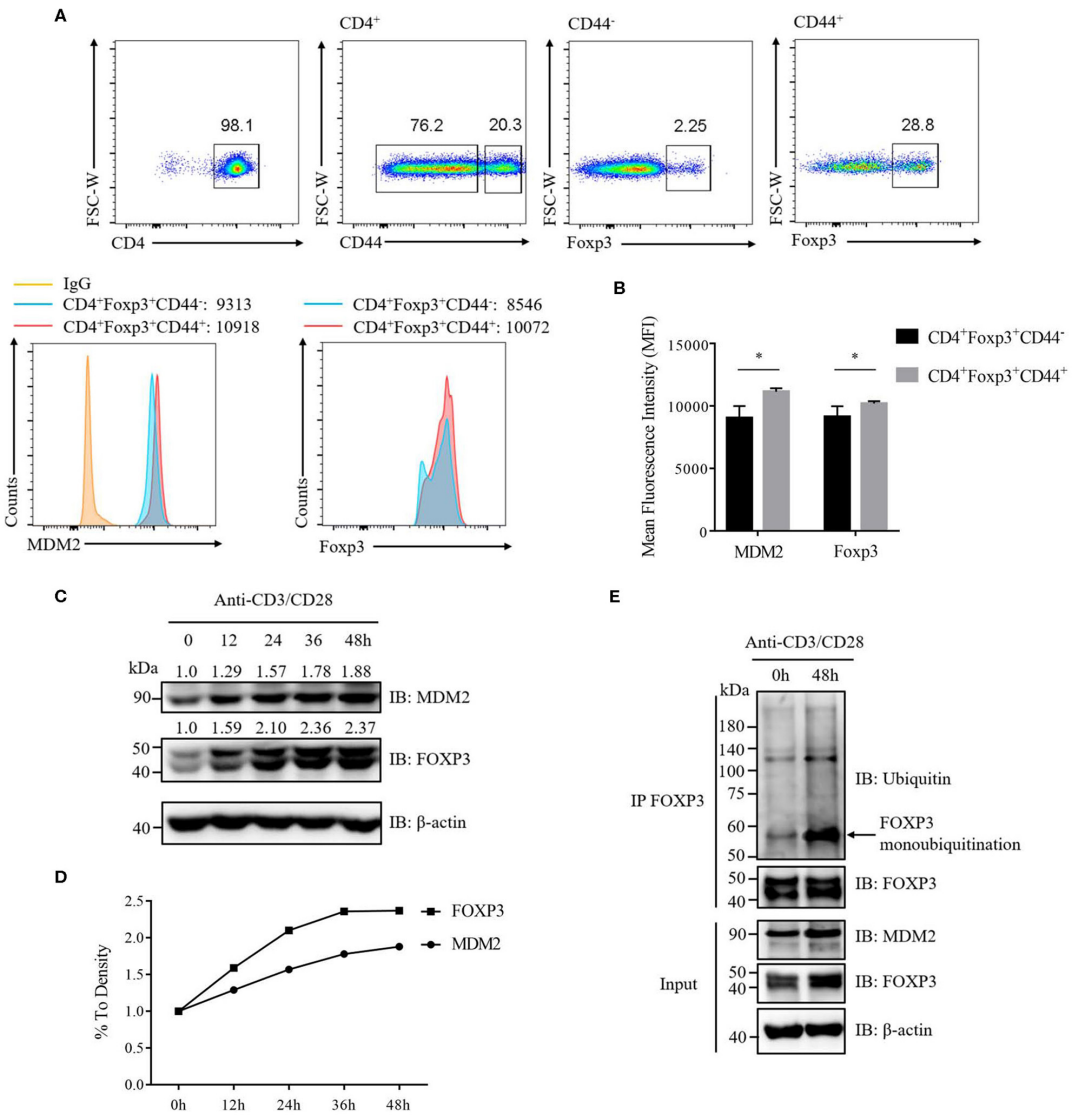
TCR/CD28 signaling enhances MDM2 expression and MDM2-mediated ubiquitination of FOXP3 in human Treg cells. **(A)** MDM2 and Foxp3 expression levels were examined in activated Treg cells (CD4^+^Foxp3^+^CD44^high^) and non-activated Treg cells (CD4^+^Foxp3^+^CD44^low^) of CD4^+^ T cells isolated from lymph nodes of WT C57BL/6 mice. **(B)** MDM2 and Foxp3 expression levels were analyzed as demonstrated in **(A)**. **(C,D)** *In vitro* differentiated human Treg cells were rested (which meant Treg cells were removed from anti-CD3/CD28 beads, with addition of 100 U/ml IL-2) for 2 days, and were re-stimulated with anti-CD3 (1 μg/ml) and anti-CD28 (1 μg/ml) antibodies in the presence of 100 U/ml IL-2 for 0, 12, 24, 36, or 48 h. The endogenous MDM2 and FOXP3 expression levels were detected by Western blot assay. **(E)** Human Treg cells were rested for 2 days and were stimulated with anti-CD3 (1 μg/ml) and anti-CD28 (1 μg/ml) antibodies in the presence of 100 U/ml IL-2 for 0 or 48 h, followed by the assessment of FOXP3 ubiquitination and FOXP3 and MDM2 expression, through utilizing immunoprecipitation assay and Western blot assay. The arrow indicates FOXP3 monoubiquitination. The above data are derived from more than three independent experiments. *p < 0.05.

To further determine the correlation between MDM2 and FOXP3 in human Treg cells, we investigated the signaling that could regulate MDM2-mediated ubiquitination modification of FOXP3. It was reported that the expression level of MDM2 was upregulated in mouse naïve CD4^+^ T cells following stimulation with anti-CD3 and anti-CD28 antibodies (30), which inspired us to explore whether TCR/CD28 stimulation may influence MDM2 expression in Treg cells. We first compared the expression levels of MDM2 and Foxp3 in activated Treg cells (CD4^+^CD44^high^Foxp3^+^) and non-activated Treg cells (CD4^+^CD44^low^Foxp3^+^) isolated from WT C57BL/6 mice, and observed that MDM2 and Foxp3 expression levels were higher in activated Treg cells (Figures 7A,B), suggesting that TCR/CD28 activation may upregulate MDM2 expression in mouse Treg cells.

To confirm whether TCR/CD28 activation could elevate MDM2 expression in human Treg cells, we stimulated these cells with anti-CD3 and anti-CD28 antibodies at a series of time points, and found that the expression levels of MDM2 and FOXP3 were upregulated along with TCR/CD28 stimulation (Figures 7C,D). To further examine whether TCR/CD28 signaling could facilitate MDM2-mediated ubiquitination modification of FOXP3, we stimulated human Treg cells with anti-CD3 and anti-CD28 antibodies for 48 h, and found that FOXP3 ubiquitination, especially monoubiquitination, as well as MDM2 and FOXP3 expression, was enhanced following TCR/CD28 activation (Figure 7E). In conclusion, these findings indicate that TCR/CD28 signaling activation enhances the expression level of MDM2 and promotes MDM2-mediated ubiquitination of FOXP3, therefore partly contributing to the elevated FOXP3 expression in human Treg cells following TCR/CD28 stimulation.

## Discussion

FOXP3 is the chief transcription factor of Treg cells and plays pivotal roles in Treg cell development and function (5–7). FOXP3 could be regulated by ubiquitination modification at the post-translational level, thus affecting FOXP3 stability and Treg cell function. In recent years, it has been found that E3 ubiquitin ligases or DUBs, including STUB1, USP7, and USP21, regulate K48-linked polyubiquitination of FOXP3, which impact FOXP3 stability and Treg cell function (17–19). However, other types of FOXP3 ubiquitination modification are largely unclear. Owing to the diverse sites and types, ubiquitination not only triggers protein degradation, but also stabilizes substrate function (21, 22). Therefore, there are new enzymes regulating FOXP3 ubiquitination, which need to be identified. Previous studies have reported that MDM2, an E3 ubiquitin ligase containing RING finger domain, mediates K48-linked polyubiquitination of p53, Rb, p21, hnRNPK, and FOXO3a proteins and degrades them through proteasome-mediated pathway (23, 24, 28, 29); however, MDM2-mediated non-degradative ubiquitination remains largely unclear. Though MDM2 has been widely studied in tumors, we understand little about whether it can regulate the function of the immune system. Recently, it has been reported that MDM2 negatively modulates T cell activation, revealing its critical role in Teff cells (30), which prompts us to investigate whether MDM2 may play an indispensable role in Treg cells. Here, we reveal that MDM2, which has been reported to mainly mediate K48-linked polyubiquitination and degradation of several substrates, could mediate non-degradative ubiquitination and facilitate the stability of FOXP3 protein, thus positively regulating human Treg cell function. Although TRAF6 has been reported to mediate K63-linked polyubiquitination of FOXP3 and facilitate mouse Treg cell function (36), our findings that MDM2 could mediate various types of ubiquitination of FOXP3 (especially monoubiquitination), thus enhancing human Treg cell function, will add greatly to human Treg cell biology.

In this study, we demonstrated that MDM2 could facilitate FOXP3 stability, thus enhancing human Treg cell function. MDM2 knockdown with shRNA-bearing lentiviruses led to reduced expression levels of CTLA-4 and CD25, while enhancing the ability to produce IL-2 and IFN-γ in human Treg cells. Since the expression of CD25, CTLA-4, IL-2, and IFN-γ is regulated by FOXP3 (31, 32), the above phenomena represented that the ability of FOXP3 to regulate downstream genes was impaired after MDM2 knockdown, which was consistent with the impaired capacity of Treg cells to inhibit Teff cell proliferation. Treg cells secreted more cytokines IL-2 and IFN-γ after the downregulation of MDM2, suggesting that these Treg cells were undergoing the transition into Teff cells, especially Th1 cells, which reflected the instability and plasticity of Treg cells (37–39). Meanwhile, Treg cells with MDM2 knockdown displayed significantly attenuated FOXP3 expression, suggesting that MDM2 knockdown led to FOXP3 instability. The above-mentioned phenotypes following MDM2 knockdown may be attributed to the instability of FOXP3 protein, therefore weakening the suppressive capacity of Treg cells. Conversely, owing to the elevated FOXP3 stability, Treg cells overexpressing MDM2 exhibited higher CD25 and CTLA-4 expression and the enhanced suppressive activity. In addition, we verified in HEK293T cells that MDM2 overexpression could improve the stability of FOXP3 protein. Therefore, we conclude that MDM2 positively regulates FOXP3 stability, thus facilitating Treg cell suppressive function.

It was reported that MDM2 could not only ubiquitinate and degrade p53 protein, but also inhibit p53 transcription process, thus negatively controlling p53 expression (25–27). We found that knocking down MDM2 in human Treg cells did not obviously affect the mRNA level of FOXP3, indicating that MDM2 may not regulate FOXP3 transcription and mainly modulate FOXP3 expression at the post-translational modification level, most likely through the ubiquitination modification of FOXP3. We confirmed that MDM2 mediated various types of ubiquitination of FOXP3, especially monoubiquitination, thus stabilizing FOXP3 expression, using HEK293T cell line overexpression system and *in vitro* ubiquitination assay. Our findings together with previous studies imply that different substrates may be regulated by MDM2 at various levels, and diverse types of ubiquitination can work together to exert similar functions.

Our results of co-expression and His-pull-down assays demonstrated that 10 FOXP3 mutants (FOXP3 K144 only, K227 only, K250 only, K263 only, K268 only, K277 only, K332 only, K382 only, K393 only, and K415 only) could be ubiquitinated and stabilized by MDM2, implicating that the above 10 lysine residues in FOXP3 are crucial for MDM2-mediated ubiquitination and stabilization of FOXP3. Among these lysine residues, K227, K250, K263, and K268 are also required for STUB1-mediated K48-linked polyubiquitination and degradation of FOXP3 (17), and K263 (its mouse equivalent, K262) is responsible for TRAF6-mediated K63-linked polyubiquitination and localization of FOXP3 (36). We therefore infer that MDM2 may have antagonistic effects against STUB1 and synergy effects with TRAF6 on the ubiquitination modification and stability of FOXP3. Since impaired TRAF6-mediated K63-linked ubiquitination leads to FOXP3 nuclear exclusion in mouse Treg cells (36), we hypothesize that MDM2 knockdown may also disrupt nuclear FOXP3 localization in human Treg cells because of the same lysine residue K263 on FOXP3. The above hypotheses need to be further explored in future studies.

Our data revealed that TCR/CD28 signaling stimulation could enhance the expression levels of MDM2 and FOXP3, and MDM2-mediated FOXP3 ubiquitination modification in human Treg cells. Although TCR/CD28 signaling activation can promote the transcription of FOXP3 and increases FOXP3 expression in Treg cells (40, 41), the elevated FOXP3 protein level may be partly due to the upregulated expression of MDM2 and its mediated FOXP3 ubiquitination modification following TCR/CD28 activation. Our findings also indicated that MDM2 and FOXP3 displayed positive correlation in human Treg cells after TCR/CD28 signaling stimulation.

Typically, Treg cells suppress the immune responses in tumor microenvironment, thus contributing to tumor progression (42). MDM2 is an extensively studied oncoprotein, and we demonstrated that it could positively regulate the suppressive function of human Treg cells. Previous studies together with our findings imply that, for one thing, MDM2 may inhibit the activity of tumor suppressor proteins (such as p53) in tumor cells; for another, it may enhance the immunosuppressive capacity of Treg cells in tumor microenvironment, thus jointly facilitating the progression of tumors. This hypothesis needs to be proved in future studies. Our findings can help us understand the relationship among MDM2, Treg cells, and tumors. Furthermore, Treg cells can suppress autoimmunity (2, 43), and MDM2 is able to regulate the immunosuppressive activity of Treg cells, which provides a novel therapeutic target for autoimmune diseases.

In summary, our work uncovers that MDM2 is crucial for positively modulating human Treg cell function and sheds light on the underlying mechanism in which MDM2 mediates the ubiquitination modification of FOXP3 and thus facilitates FOXP3 stability.

## Materials and Methods

### Plasmids

MDM2 was amplified from HEK293T cell cDNA and was cloned into PIP-Flag-tagged vectors. PIP-Flag-tagged MDM2 mutants C478S and C464A were constructed by site-directed mutagenesis and were confirmed by sequencing. PIP-Myc-tagged FOXP3, PIP-HA-tagged FOXP3 and mutants, and PIP-6 × his-tagged Ubiquitin and mutants were constructed as described previously (17).

### Antibodies

These antibodies were used in this study for immunoprecipitation (IP), Western blot (WB), flow cytometry (FACS), and immunofluorescence (IF): Mouse anti-Flag (Sigma-Aldrich F3165, WB 1:10,000, IP 1 μg/ml), Mouse anti-HA (Santa Cruz Biotechnology sc-7392, WB 1:1,000), Mouse anti-Myc (Santa Cruz Biotechnology sc-40, WB 1:1,000, IP 1 μg/ml), Mouse anti-MDM2 (Sigma-Aldrich M4308, WB 1:1,000, IP 1 μg/ml, FACS or IF 1:200), Mouse anti-FOXP3 (eBioscience eBio7979 14-7979-82, WB 1:2,500, IP 1 μg/ml), Rabbit anti-FOXP3 (Abcam ab54501, WB 1:4,000, IF or IP 1 μg/ml), Mouse anti-Ubiquitin (Santa Cruz Biotechnology sc-8017, WB 1:1,000), Mouse anti-β-actin (Proteintech 60008-1-Ig, WB 1:10,000), Mouse IgG1 isotype control (CST 5415, IP, IF or FACS 1 μg/ml), Normal rabbit IgG (CST 2729, IP, IF or FACS 1 μg/ml), Goat anti-mouse IgG light chain (Jackson Immuno Research 115-035-174, WB 1:2,000), Anti-mouse IgG-HRP conjugate (Promega W4021, WB 1:5,000), Goat anti-rabbit IgG light chain (Jackson Immuno Research 211-002-171, WB 1:2,000), Goat anti-rabbit IgG, HRP-linked antibody (CST 7074, WB 1:3,000), Goat anti-mouse IgG Alexa Fluor 488 (Thermo Fisher Scientific A11029, IF or FACS 1:1,000), Goat anti-mouse IgG Alexa Fluor 555 (Thermo Fisher Scientific A32727, IF 1:1,000), Goat anti-rabbit IgG Alexa Fluor 488 (Thermo Fisher Scientific A11008, IF 1:1,000), and DAPI (4,6-diamidino-2-phenylindole, dihydrochloride, Invitrogen D1306, IF 1:1,000). These antibodies were used for flow cytometry only: PE-Cy7 anti-human CD152 (CTLA-4) (Biolegend 349914, 1:200), PE anti-human CD25 (BD Pharmingen 555432, 1:200), Anti-human FOXP3 APC (eBioscience 17-4777-42, 1:200), Anti-human IFN-γ APC (eBioscience 17-7319-82, 1:200), PE anti-human IL-2 (BD Pharmingen 559334, 1:200), APC anti-human CD4 (Biolegend 317416, 1:200), PE-Cy7 anti-human CD4 (Biolegend 317413, 1:200), PE anti-human CD4 (BD Pharmingen 555347, 1:200), FITC anti-human CD4 (BD Pharmingen 555346, 1:200), CD44-PE, mouse (Miltenyi Biotec 130-102-606, 1:200), Anti-mouse/rat Foxp3 APC (eBioscience 17-5773-82, 1:200), Anti-mouse CD4 PerCP-Cy5.5 (eBioscience 45-0042-82, 1:200), Anti-mouse CD4 PE (eBioscience 12-0042-85, 1:200), and Anti-mouse CD4 APC (eBioscience 17-0041-83, 1:200). Flow cytometry data were collected on BD LSRFortessa or LSRFortessa X-20, and analyzed using FlowJo software.

### Quantitative Real-Time PCR (qRT-PCR)

Total RNA was extracted from human Treg cells using TRIzol reagent (Invitrogen), according to the instructions of the manufacturer. RNA was quantified, and was reverse-transcribed into cDNA using the PrimeScript RT reagent kit (TaKaRa, RR037A). PCR reactions were performed with SYBR Premix Ex Taq reagent (TaKaRa, RR420A) on the ABI 7900HT Sequence Detection System. The relative target mRNA expression levels were normalized to the expression of β-actin.

The following primers were used for qRT-PCR:

MDM2-human-Forward: 5′-GGCAGGGGAGAGTGATACAGA-3′,
MDM2-human-Reverse: 5′-GAAGCCAATTCTCACGAAGGG-3′,
FOXP3-Human-Forward: 5′-TCCCAGAGTTCCTCCACAAC-3′,
FOXP3-Human-Reverse: 5′-ATTGAGTGTCCGCTGCTTCT-3′,
β-Actin-Human-Forward: 5′-CTCTTCCAGCCTTCCTTCCT-3′,
β-Actin-Human-Reverse: 5′-CAGGGCAGTGATCTCCTTCT-3′.

### Cell Culture and Transfection

HEK293T cells were grown in Dulbecco's modified Eagle's medium (DMEM) (Gibco) supplemented with 10% fetal bovine serum (FBS, Gibco) and 1% Penicillin–Streptomycin (Gibco 15140122). Jurkat T cells were cultured in PRMI 1640 medium (Gibco) with addition of 10% FBS, 1% GlutaMAX (Gibco 35050061), 1% Sodium Pyruvate (Gibco 11360070), 1% MEM Non-Essential Amino Acids Solution (Gibco 11140050), and 1% Penicillin–Streptomycin. All cells were cultured in a 37°C/5% CO_2_ incubator. HEK293T cells were transfected with plasmids using polyethylenimine (PEI, Polysciences 23966-2), following the manufacturer's instructions.

### Isolation of Naïve CD4^+^ T Cells, Treg Cells, and Teff Cells

Human peripheral blood mononuclear cells (PBMCs) were isolated from blood samples of healthy donors from Shanghai Blood Center, which was approved by Shanghai Blood Center Ethics Committee.

Human total CD4^+^ T cells were isolated from human PBMCs using human CD4 MicroBeads (Miltenyi Biotec 130-045-101). Human naïve CD4^+^ T cells (CD4^+^CD25^low^CD127^high^CD45RA^+^), Treg cells (CD4^+^CD25^high^CD127^low^), and Teff cells (CD4^+^CD25^low^CD127^high^) were sorted by BD FACS AriaII cell sorter. The purity of the above-sorted cells was at least 90%. Human naïve CD4^+^ T cells were differentiated into induced Treg cells (iTreg cells) in X-VIVO 15 medium (Lonza 04-418Q) containing 10% FBS (Gibco 10100147), 1% GlutaMAX, 1% Sodium Pyruvate, 1% MEM Non-Essential Amino Acids Solution, 1% Penicillin–Streptomycin, 100 U/ml IL-2 (R&D Systems 202-IL), and 5 ng/ml TGF-β (R&D systems 240-B), in the presence of Dynabeads Human T-activator CD3/CD28 (Gibco 1132D) at a bead-to-cell ratio of 1:2. After about 7 days, the differentiation efficiency was at least 90%. Human Treg cells were expanded in the above X-VIVO 15 complete medium in the presence of Dynabeads Human T-activator CD3/CD28 and 500 U/ml IL-2. Human Teff cells were expanded in the above X-VIVO 15 complete medium in the presence of Dynabeads Human T-activator CD3/CD28 and 50 U/ml IL-2.

Mouse total CD4^+^ T cells were isolated from lymph nodes using mouse CD4 (L3T4) MicroBeads (Miltenyi Biotec 130-049-201) and were cultured in PRMI 1640 medium supplemented with 10% FBS (Gibco 10100147), 1% GlutaMAX, 1% Sodium Pyruvate, 1% MEM Non-Essential Amino Acids Solution, and 1% Penicillin–Streptomycin.

### Lentivirus Construction and Infection

For knockdown assay, MDM2 shRNAs or shCK control were cloned into PLKO.1 lentiviral vectors with green fluorescent protein (GFP). PLKO.1-shMDM2-GFP or PLKO.1-shCK-GFP, Δ8.9, and vesicular stomatitis virus glycoprotein (VSVG) were co-transfected into HEK293T cells with PEI reagent. MDM2 shRNA target sequences were as follows: shMDM2-1, 5′-GGAGCAGGCAAATGTGCAA-3′; shMDM2-2, 5′-GGAACTTGGTAGTAGTCAA-3′; shMDM2-3, 5′-GCACTTCATGCAATGAAAT-3′. Scrambled shRNA was used as shCK control, and the target sequence was 5′-TTCTCCGAACGTGTCACGT-3′.

For overexpression assay, MDM2 coding sequence (CDS) was cloned into PLVX lentiviral vectors with GFP. PLVX-MDM2-GFP or PLVX-CK-GFP, Δ8.9, and VSVG were co-transfected into HEK293T cells with PEI. The supernatants containing viruses were harvested after 48 h and were purified and enriched via filter and ultracentrifugation.

Before infection, human iTreg or Teff cells were expanded for 7 days and were stimulated with anti-CD3/anti-CD28 antibodies (1 μg/ml) for 48 h. Next, iTreg or Teff cells were infected with lentiviruses, in the presence of IL-2 (100 U/ml for iTreg cells and 50 U/ml for Teff cells) and 8 μg/ml polybrene (Sigma-Aldrich H9268) overnight. Jurkat T cells were grown to proper amounts and were infected with lentiviruses, in the presence of 8 μg/ml polybrene overnight. Then, cell supernatants containing lentiviruses were replaced with fresh medium. Four days post-infection, viable GFP^+^ cells were sorted by BD FACS AriaII or detected by flow cytometry.

### *In vitro* Suppression Assay

Human Teff cells (CD4^+^CD25^low^CD127^high^) were sorted from healthy donor PBMCs using BD FACS AriaII and were labeled with 5 μM CellTrace Violet (CTV, Invitrogen C34557) for 20 min at 37°C. Human *in vitro* expanded Treg cells or iTreg cells were diluted at various concentrations and were incubated with Teff cells that were labeled with CTV and activated with Dynabeads Human T-activator CD3/CD28 (Teff cells: beads = 4:1) in a U-bottom 96-well-plate. Ninety hours later, cells were harvested, incubated with Viability Dye eFluor 780 (eBioscience 65-0865-14), and examined for Teff cell proliferation on BD LSRFortessa X-20.

### Immunoprecipitation and Western Blot

Cells were harvested, washed with 1 × PBS, and lysed at 4°C for 30 min in radio immunoprecipitation assay buffer (RIPA buffer) (50 mM Tris–HCl, pH 7.5, 135 mM NaCl, 0.5% sodium deoxycholate, 10% glycerol, 1 mM EDTA, and 0.5% Nonidet P-40) supplemented with 1% proteinase inhibitor cocktail (sigma-Aldrich P8340), 1 mM PMSF, 1 mM NaF, and 1 mM Na_3_VO_4_. Cell lysates were centrifugated, and the supernatants were immunoprecipitated with 1 μg of the corresponding antibodies at 4°C for 3 h or overnight, followed by incubation with 10 μl of protein A/G Plus-agarose (Santa Cruz Biotechnology sc-2003) at 4°C for 1 h. The immunocomplexes were washed six times with RIPA buffer containing 1 mM PMSF. Then samples were detected by Western blot assay according to standard procedures.

### His-Pull-Down Assay

HA-tagged FOXP3, Flag-tagged MDM2, and His-tagged ubiquitin (Ubi) or mutants were co-transfected into HEK293T cells. About 48 h post-transfection, cells were harvested, washed with 1 × PBS, and lysed at room temperature for 30 min in Buffer 1 (8 M urea, 100 mM Na_2_HPO_4_, 10 mM Tris–HCl, pH 8.0, 0.2% Triton X-100, and 10 mM imidazole). The lysates were incubated with nickel-nitrilotriacetic acid beads (Ni-NTA Beads, Qiagen 30210) at room temperature for 3 h. During the incubation, beads were washed three times by Buffer 1. After incubation, beads were washed twice in Buffer 1, twice in Buffer 2 (8 M urea, 100 mM Na_2_HPO_4_, 10 mM Tris–HCl, pH 6.3, 0.2% Triton X-100, and 10 mM imidazole), and once in Buffer 3 [100 mM NaCl, 20 mM Tris–HCl, pH 8.0, 20% glycerol, 1 mM dithiothreitol (DTT), and 10 mM imidazole]. The ubiquitination levels of HA-tagged FOXP3 were assessed by Western blot assay with anti-HA antibodies.

### *In vitro* Ubiquitination Assay

6 × his-E1, 6 × his-UbcH5b (E2), 6 × his-Flag-ubiquitin, and StrepII-FOXP3a were purified as previously described (17, 20). GST-MDM2 recombinant protein was bought from R&D Systems (E3-202). 6 × his-E1 (400 ng), 6 × his-UbcH5b (E2) (800 ng), 6 × his-Flag-ubiquitin (12 μg), StrepII-FOXP3a (400 ng), and GST-MDM2 (1.5 μg) were incubated at 37°C in a cell-free system (20 mM Tris–HCl, pH 7.5, 5 mM MgCl_2_, 5 mM DTT, and 2 mM ATP) for 2 h, followed by termination with sodium dodecyl sulfate (SDS) loading buffer. FOXP3 ubiquitination was examined by Western blot assay with anti-FOXP3 antibodies.

### Immunofluorescence Assay

Immunofluorescence assay was performed as previously described (20).

### C57BL/6 Mice

WT mice were purchased from the Jackson Laboratory and were bred under specific pathogen-free condition. All mouse protocols were approved by the Institutional Animal Care and Use Committee in Institut Pasteur of Shanghai, CAS.

### Statistical Analysis

The significant differences of statistical data were determined by two-tailed, unpaired Student's *t-*test using GraphPad Prism 7. All data represent means ± SD. Unless otherwise indicated, the data are derived from more than three independent experiments. The *p* < 0.05 was considered statistically significant: ^*^p < 0.05, ^**^p < 0.01, ^***^p < 0.001; ns, not significant. Relative quantification of Western blot results was assessed by Image J software.

## Data Availability Statement

The datasets generated for this study are available on request to the corresponding author.

## Ethics Statement

The animal study was reviewed and approved by The Institutional Animal Care and Use Committee in Institut Pasteur of Shanghai, CAS.

## Author Contributions

AW designed and performed experiments and drafted the manuscript. MY and RLia analyzed and interpreted the data. FaZ, FuZ, and XL gave technical advice. YH, RLin, and XW contributed to experimental ideas. DL, HL, XY, and HZ revised the manuscript. BL revised the manuscript and provided overall direction. All authors contributed to the article and approved the submitted version.

## Conflict of Interest

BL is a co-founder of Biotheus Inc. and chairman of its scientific advisory board. The remaining authors declare that the research was conducted in the absence of any commercial or financial relationships that could be construed as a potential conflict of interest.
