# Supplementary material for: Mouse Double Minute 2 Homolog-Mediated Ubiquitination Facilitates Forkhead Box P3 Stability and Positively Modulates Human Regulatory T Cell Function
Source: Front Immunol. 2020 Jun 19;11:1087. doi: 10.3389/fimmu.2020.01087 (PMC7318079; doi:10.3389/fimmu.2020.01087)
Supplement: Supplementary file 1 [file Data_Sheet_1.docx]

Supplementary Material

**
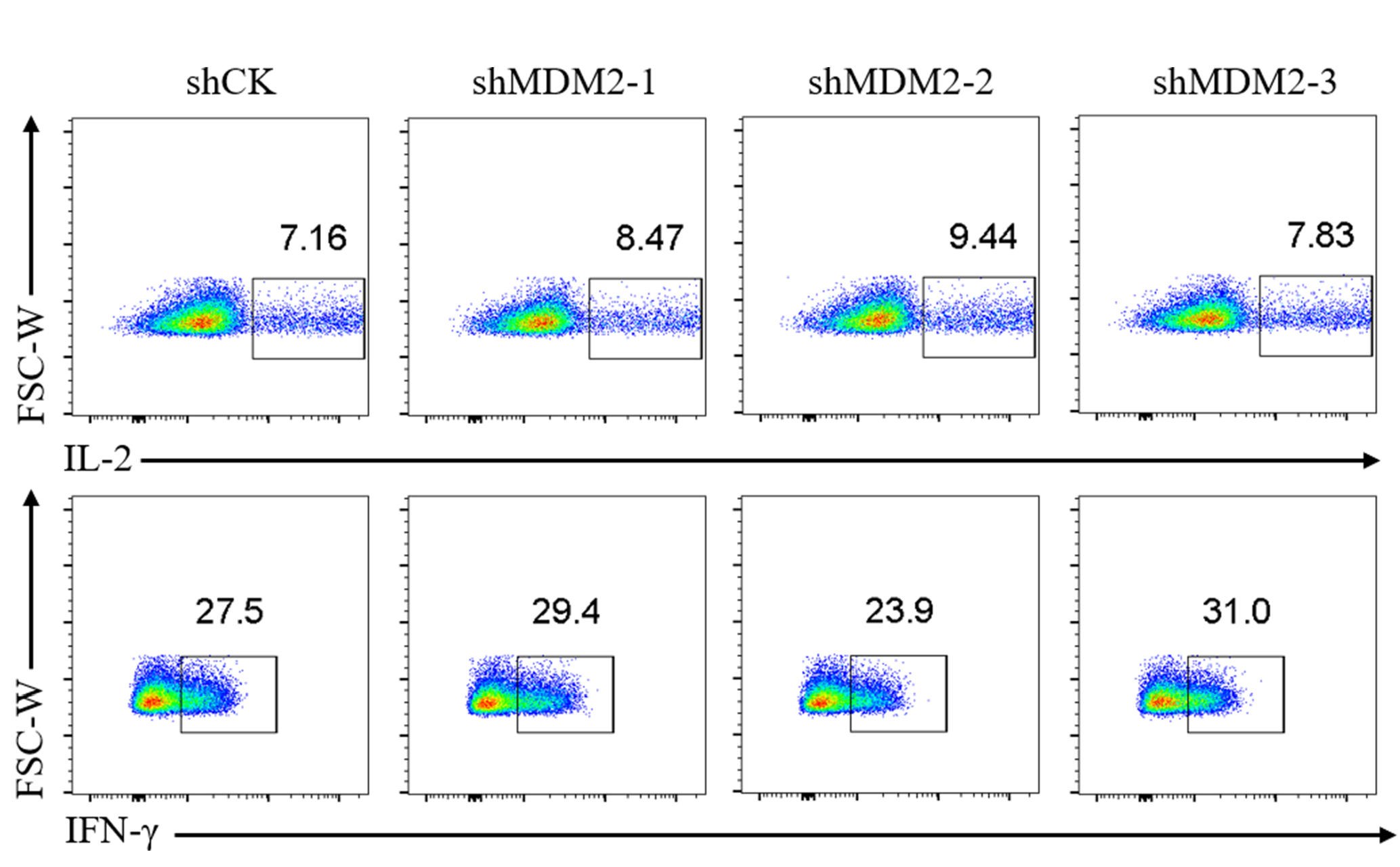
**

**Supplementary Figure 1. MDM2 knockdown does not affect IL-2 and IFN-γ production from human Teff cells.** MDM2 was knocked down in human *in vitro* expanded Teff cells (CD4^+^CD127^high^CD25^low^) using MDM2 shRNA-carrying lentiviruses, followed by flow cytometry analysis for IL-2 and IFN-γ production (n=3).

**
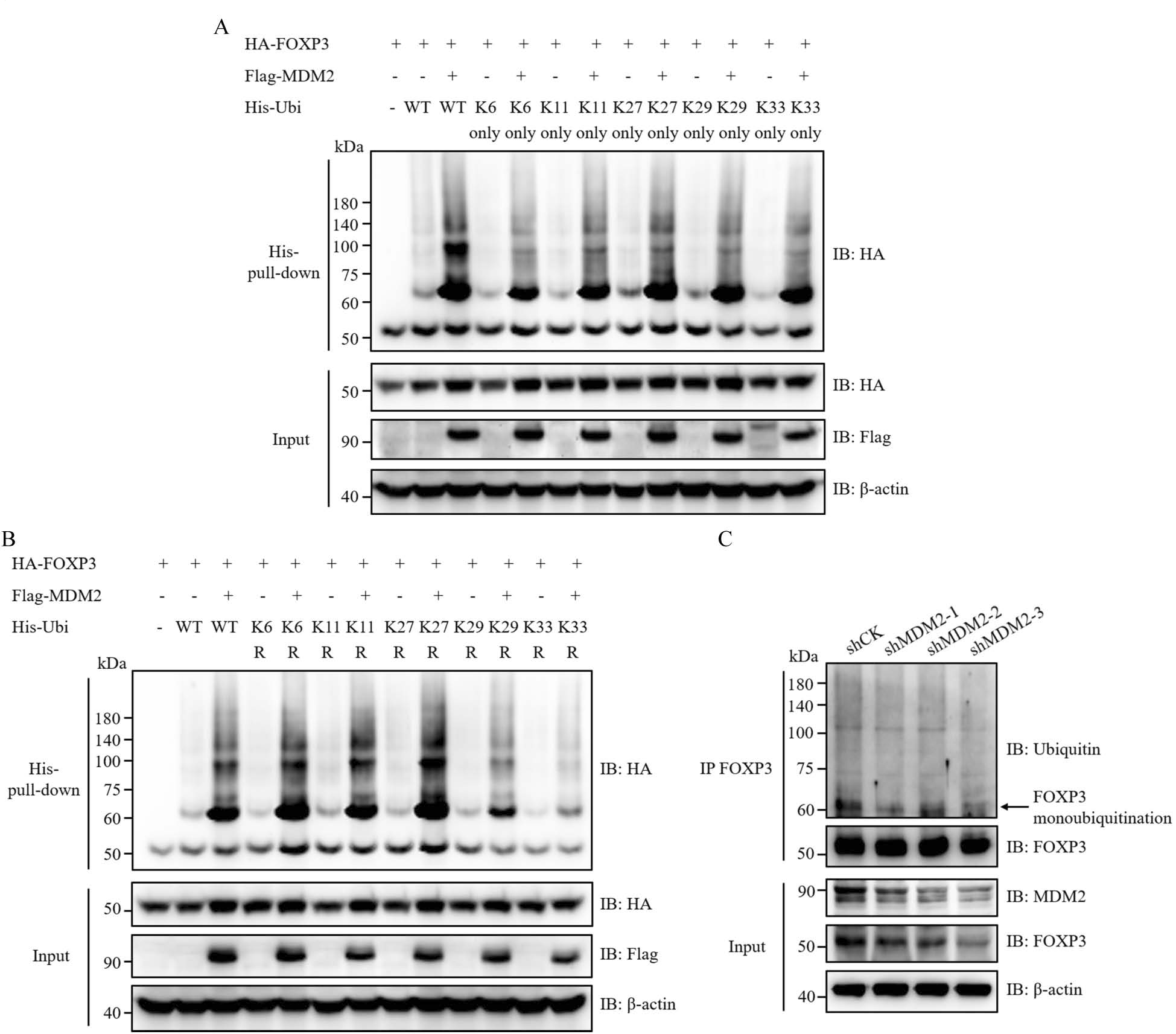
**

**Supplementary Figure 2. MDM2 mediates ubiquitination of FOXP3 which facilitates FOXP3 stability.** **(A)** HA-tagged FOXP3, Flag-tagged MDM2, and His-tagged WT ubiquitin (Ubi), or ubiquitin mutants (with conservation of only one lysine residue) were co-transfected into HEK293T cells. 48 hours post-transfection, the levels of FOXP3 ubiquitination were assessed by His-pull-down assay and western blot assay. **(B)** HA-tagged FOXP3, Flag-tagged MDM2, and His-tagged WT ubiquitin, or ubiquitin mutants (with one lysine-to-arginine mutation) were co-transfected into HEK293T cells, and 48 hours post-transfection, the levels of FOXP3 ubiquitination were examined by His-pull-down assay and western blot assay. **(C)** FOXP3 ubiquitination was detected in Flag-FOXP3 Jurkat T cells infected with lentiviruses carrying MDM2 shRNA or shCK vectors, by immunoprecipitation assay and western blot assay. The arrow implies FOXP3 monoubiquitination. The above data are derived from more than three independent experiments.


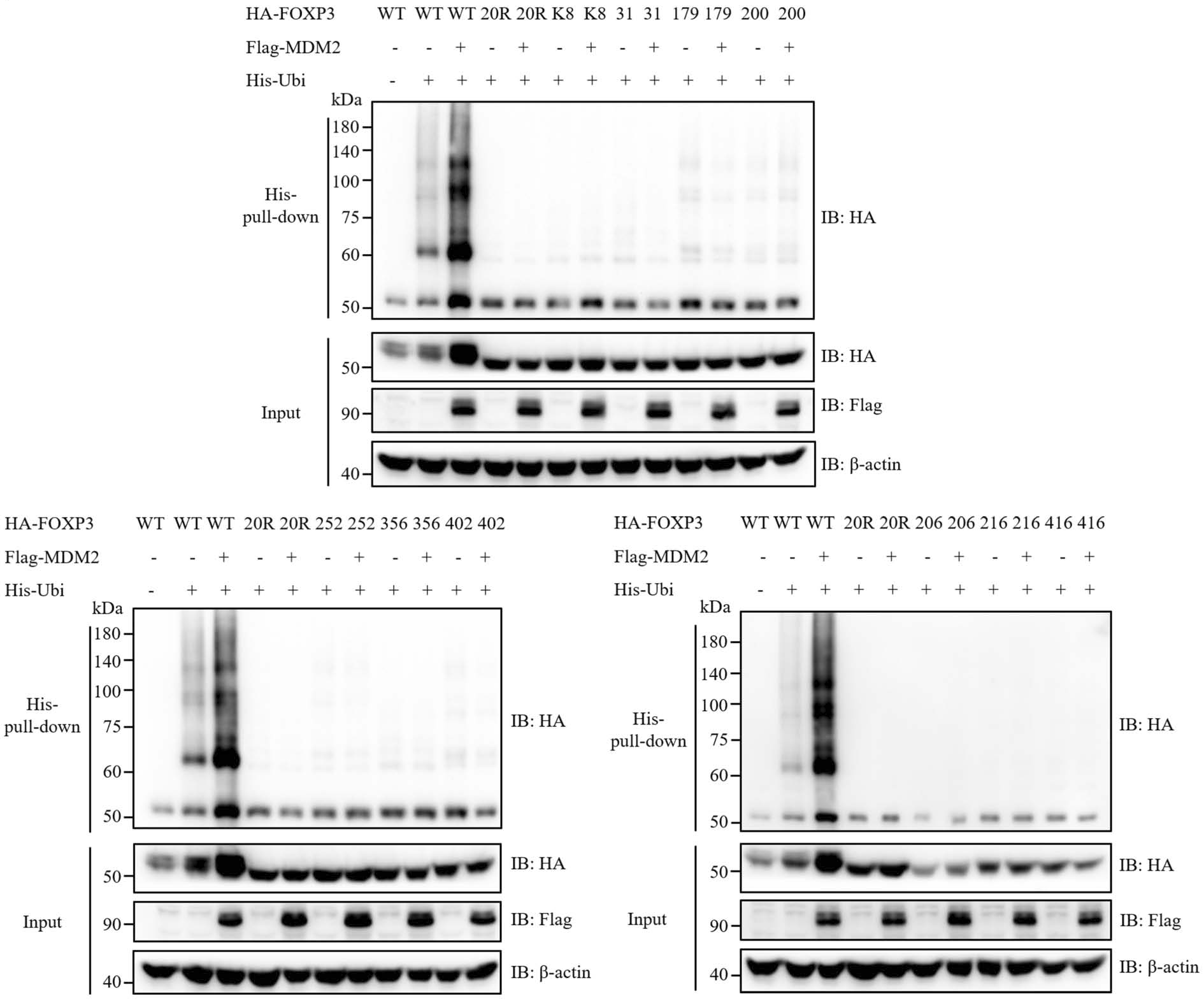


**Supplementary Figure 3.** **Ten lysine residues in FOXP3 are not required for MDM2-mediated ubiquitination.** HA-tagged WT FOXP3 or FOXP3 mutants, Flag-tagged MDM2 and His-tagged ubiquitin were co-transfected into HEK293T cells, and 48 hours post-transfection, His-pull-down assay was performed to assess the ubiquitination levels of HA-FOXP3. Ten FOXP3 mutants (K8 only, K31 only, K179 only, K200 only, K206 only, K216 only, K252 only, K356 only, K402 only, K416 only) could not be ubiquitinated and stabilized by MDM2. The above data are derived from more than three independent experiments.
